# Modeling the effect of rainfall changes to predict population dynamics of the Asian tiger mosquito *Aedes albopictus* under future climate conditions

**DOI:** 10.1371/journal.pone.0268211

**Published:** 2022-05-25

**Authors:** Shin Fukui, Yusuke Kuwano, Kazuki Ueno, Kazuyuki Atsumi, Shunji Ohta

**Affiliations:** 1 Department of Human Behavior and Environment Sciences, Faculty of Human Sciences, Waseda University, Tokyo, Japan; 2 Fisheries Data Sciences Division, Fisheries Stock Assessment Center, Fisheries Resources Institute, Japan Fisheries Research and Education Agency, Yokohama, Japan; 3 Department of Evolutionary Studies of Biosystems, School of Advanced Sciences, SOKENDAI (The Graduate University for Advanced Studies), Hayama, Japan; ICAR-Central Insitute for Cotton Research, INDIA

## Abstract

The population dynamics of mosquitoes in temperate regions are not as well understood as those in tropical and subtropical regions, despite concerns that vector-borne diseases may be prevalent in future climates. *Aedes albopictus*, a vector mosquito in temperate regions, undergoes egg diapause while overwintering. To assess the prevalence of mosquito-borne diseases in the future, this study aimed to simulate and predict mosquito population dynamics under estimated future climatic conditions. In this study, we tailored the physiology-based climate-driven mosquito population (PCMP) model for temperate mosquitoes to incorporate egg diapauses for overwintering. We also investigated how the incorporation of the effect of rainfall on larval carrying capacity (into a model) changes the population dynamics of this species under future climate conditions. The PCMP model was constructed to simulate mosquito population dynamics, and the parameters of egg diapause and rainfall effects were estimated for each model to fit the observed data in Tokyo. We applied the global climate model data to the PCMP model and observed an increase in the mosquito population under future climate conditions. By applying the PCMP models (with or without the rainfall effect on the carrying capacity of the *A*. *albopictus*), our projections indicated that mosquito population dynamics in the future could experience changes in the patterns of their active season and population abundance. According to our results, the peak population number simulated using the highest CO_2_ emission scenario, while incorporating the rainfall effect on the carrying capacity, was approximately 1.35 times larger than that predicted using the model that did not consider the rainfall effect. This implies that the inclusion of rainfall effects on mosquito population dynamics has a major impact on the risk assessments of mosquito-borne diseases in the future.

## 1. Introduction

The climate change-induced spread of vector-borne diseases that threaten human health is a growing global concern [1]. Predicting the impact of climate change on vector-borne diseases can be problematic as vector activity is strongly influenced by habitat conditions. In the case of mosquito-borne diseases, changes in temperature, rainfall, and humidity influence the scale of infection in complex ways [2]. One of the reasons for this is that the life cycle and physiological responses of mosquitoes are dependent upon the environmental conditions of their habitat [3]. For example, the development rate and mortality of mosquitoes vary depending on the temperature of the habitat [4–7], and precipitation affects their population density during their aquatic life stages [8–12]. Therefore, the spread of mosquito-borne diseases is closely correlated with meteorological factors. In particular, a rise in temperature and precipitation due to climate change is anticipated in temperate regions [13]. There are many cities globally that have temperate-climate zones and large populations. Accordingly, the way in which climate change affects prevailing mosquito-borne infectious diseases is of great concern [14–18].

The outbreak of dengue fever was observed in the metropolis of Tokyo, one of the world’s most populous cities in the temperate climatic zone in 2014 [19]. The prediction of dengue fever in populous cities is a matter of urgent concern, not only for the area where the outbreak occurred but also for other places in the world, due to the risk of a secondary outbreak via those infected.

The population dynamics of the *Aedes* genus, a vector of dengue virus, have been previously modeled [20–25], owing to the fact that an increase in mosquito population may result in increased transmission of the dengue virus. In particular, the population model of *Aedes aegypti*, whose species are distributed in the tropics and subtropics, has been well studied [20, 21, 23], as many mosquito-borne diseases, including dengue fever, Zika, and chikungunya, originated in tropical regions. Recently, several population models for the Asian tiger mosquito (*Aedes albopictus*), which are distributed across wide climatic regions (from tropical to temperate), have been proposed [17, 22, 24, 26, 27]. The life cycle of *Aedes albopictus*, which inhabits temperate regions, has an egg diapause stage. Accordingly, Tran et al. and Jia et al. developed population models that incorporated this stage based on observational data that assumed the given diapause period fitting to the latitude of the observation area [22, 24]. For *Culex pipiens*, which has a life cycle that features an adult diapause stage [28], the physiology-based climate-driven mosquito population (PCMP) model was developed in which the diapause behavior was estimated to fit the observed data in Tokyo. Notably, in the PCMP model, the diapause is assumed to be controlled solely by the photoperiod. However, the length of mosquito’s active period is affected by other environmental factors, including habitat temperature [29–32]. Thus, future studies that use mosquito population model must incorporate diapause and habitat environment information.

Accounting for the effect of rainfall on mosquito populations within the model is crucial for the prediction of population dynamics because water plays a pivotal role in controlling the habitat conditions for juveniles [12]. Rainfall creates or expands the habitat of mosquitoes in the aquatic stage [8, 10, 11], and has a positive effect on mosquito populations. Several studies have incorporated this effect into mosquito population models as a key factor in controlling population size [24, 33, 34]. Abiodun et al. incorporated the effect of rainfall on the carrying capacity of puddles, which act as habitats for mosquito larvae, considering puddle evaporation and infiltration [34]. To predict mosquito populations under the conditions that may result from climate change, the model should simultaneously consider the effects of temperature and rainfall, since habitat size is determined by several environmental variables.

To reveal the potential risk of a dengue outbreak in Tokyo under future climatic conditions, with an emphasis on the effect of precipitation, we projected the population dynamics of *Ae*. *albopictus* using a climate-driven mechanistic population model. We improved the PCMP model [28] by including temperature and changes in photoperiod, both of which affect the diapause stage. This was modified to adjust to *Aedes* species, which are known to have a life cycle of egg diapause.

Our model has the ability to provide estimations, while considering the effects of precipitation on mosquito populations. It can predict the pattern of mosquito population dynamics under the effects of future climatic conditions and help illustrate the importance of incorporating precipitation into predictive models in temperate zones. To begin with, we confirmed the reproducibility of the observed adult female mosquito population, and subsequently used PCMP in combination with a global climate model (GCM), while considering two representative concentration pathway (RCP) scenarios. Furthermore, we demonstrated how the rainfall effect on population dynamics affects the predictions of future population and active seasons of *Ae*. *albopictus*.

## 2. Materials and methods

### 2.1 Model framework

**Fig 1:**
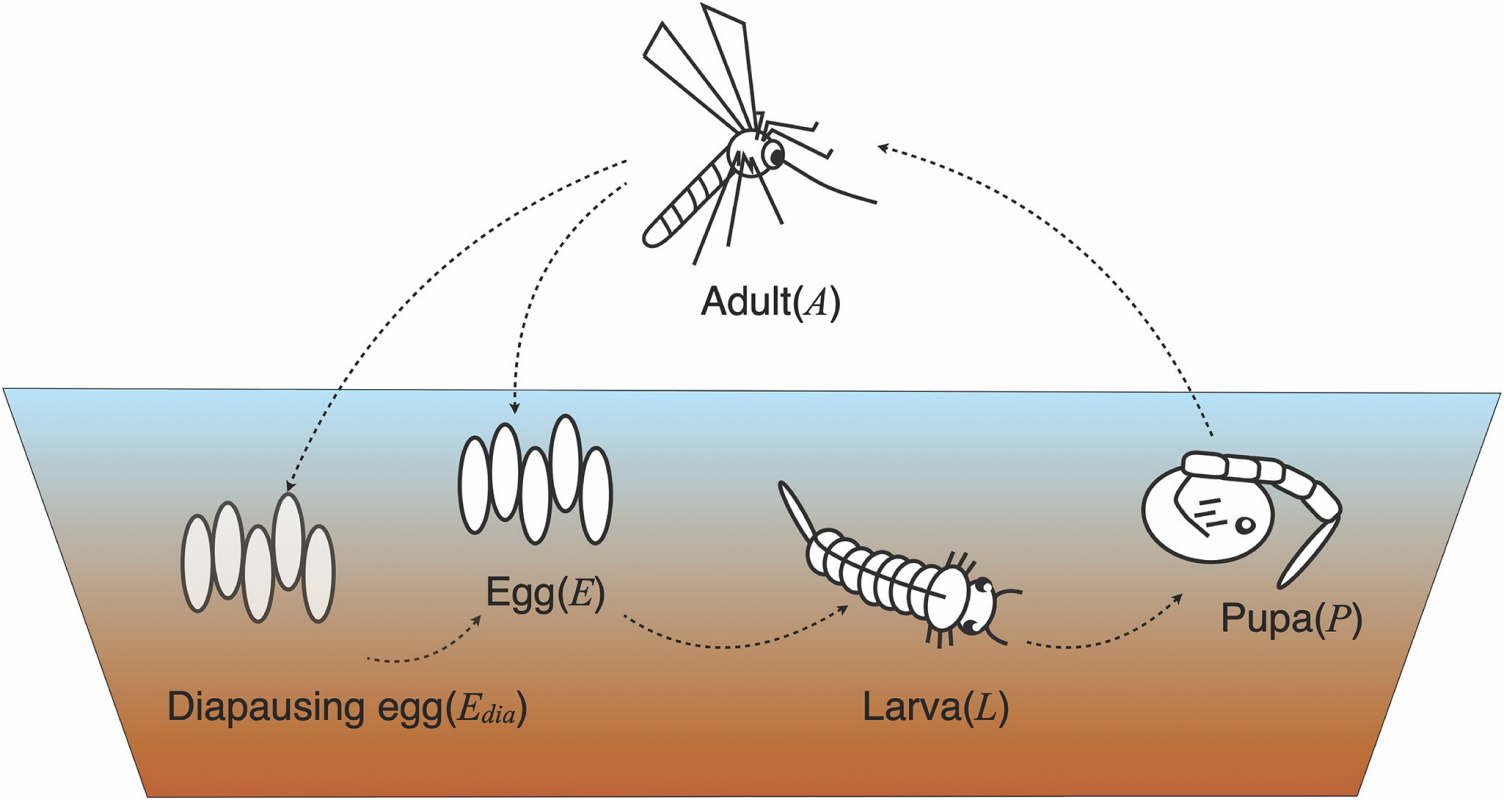
Framework of physiology-based climate-driven mosquito population (PCMP) model for *Aedes albopictus*.

The overall flow of the PCMP model, combined with the habitat meteorological data for *Ae*. *albopictus*, is shown in Fig 1. The model consists of two major parts: the habitat environment derived from microclimatic conditions, and mosquito population dynamics. The habitat environment restricts the mosquito population (Fig 1), and the thermal conditions of the habitat drives mosquito population dynamics, with water temperature affecting the aquatic stage and air temperature affecting the aerial stage.

### 2.2 Population dynamics

To formulate the population dynamics of *Ae*. *albopictus*, we modeled the development and mortality of the five life stages of this species: egg, diapause, larva, pupa, and adult, which are indicated as *E*, *E*_dia_, *L*, *P*, and *A*, respectively. These dynamics were calculated using the following equation:

$$\begin{array}{l} \dot{E}=\left(1-z_{1}\right)o_{v}A-\left(m_{E}+d_{E}\right)E+z_{2}E_{dia}\\ {\dot{E}}_{dia}=z_{1}o_{v}\;A-z_{2}E_{dia}\\ \dot{L}=d_{E}E-\left(m_{L}+d_{L}+\frac{L}{K}\right)L\\ \dot{P}=d_{L}L-\left(m_{P}+d_{P}\right)P\\ \dot{A}=d_{P}P-m_{A}A \end{array}$$ (1)

where *d*_i_ (i = *E*, *L*, and *P*), *m*_i_ (i = *E*, *L*, *P*, and *A*), *K*, *o*_*v*_, and *z*_j_ (j = 1, 2) represent the temperature-dependent development rate, temperature-dependent mortality rate, carrying capacity of larvae, temperature-dependent oviposition rate, and parameters for adult diapausing, respectively. These parameters are in daily time scales, as shown in Table 1 [24, 35, 36].

**Table 1:** Parameters used in this study, along with their functions.

Parameter	Description	Reference
*d*_i_	Temperature dependent developmental rate (i = *E*, *L*, and *P*)	[24]
*m*_i_	Temperature dependent mortality rate (i = *L*, *P* and *A*)	[24]
*m*_E_	Temperature dependent egg mortality rate	[35]
*o*_*v*_	Temperature dependent oviposition rate	[36]
*z*_1_	Probability of temperature and photoperiod dependent diapausing-egg laying	[28]
*z*_2_	Probability of temperature and photoperiod dependent diapause breaking	[28]
*W*	Soil water content [mm]	[37]
W*	Soil water-holding capacity [mm]	[38]

In this model, we used the temperature-dependent oviposition rate of *Ae*. *aegypti* and the temperature-dependent mortality rate of the egg stage of *C*. *pipiens*. The adult mosquito produces eggs and diapause eggs with probability *z*_1_ and 1−*z*_1_, and the probability of the diapause egg becoming active is *z*_2_. These probabilities can be expressed as follows:

$$\begin{array}{l} z_{1}={\left\{1+e^{-\alpha_{d1}\left(\beta_{d1}-D_{week}\right)}\right\}}^{-1}{\left\{1+e^{-\alpha_{t1}\left(\beta_{t1}-Ta_{week}\right)}\right\}}^{-1}\;where\;\delta D<0\\ z_{2}={\left\{1+e^{\alpha_{d2}\left(\beta_{d2}-D_{week}\right)}\right\}}^{-1}{\left\{1+e^{\alpha_{t2}\left(\beta_{t2}-Tw_{week}\right)}\right\}}^{-1}\;where\;\delta D>0 \end{array}$$ (2)

where *α*_Xi_, *β*_Xi_ (i = 1, 2), *δD*, *D*_week_, Ta_week_, and *Tw*_week_ are the slope and the half-saturation point of the sigmoidal curve for the photoperiod (X = d) and temperature (X = *t*) thresholds, change in daily photoperiod, average sunlight duration (h) during the most recent 7 days, average daily air temperature (°C) during the most recent 7 days, and the average daily water temperature (°C) during the most recent 7 days, respectively.

The effect of habitat water conditions on the mosquito population was examined to determine the larval carrying capacity of the species. We assumed that soil moisture altered the larval carrying capacity as it determines the larval habitat; the response of larval carrying capacity to soil moisture is non-linear, indicating that carrying capacity increases with increasing soil moisture. In the model, *K* is expressed as follows:

$$K=\kappa_{max}{\left(\frac{W}{W^{*}}\right)}^{\nu }$$ (3)

where *κ*_*max*_, *W*, and *v* represent the maximum carrying capacity, soil water content (mm), and the responsiveness of carrying capacity to soil moisture, respectively. W* refers to the soil water-holding capacity (mm), which reflects the effects of soil texture, soil organic content, and plant root depth, obtained from [38]. The soil moisture ratio (%) was derived from *W*/W*.

### 2.3 Field observations, parameter estimation, and meteorological data

We used the mosquito field observation data collected by Tsuda and Hayashi containing details of the number of adult mosquitoes captured from one site using two traps set at different heights from the ground, which was collected on a weekly basis from May 2003 to December 2013 at the National Institute of Infectious Disease in Tokyo, Japan [39]. This field survey was conducted using a dry-ice trap, which implies that the captured mosquitoes were female, and they are responsible for the transmission of several mosquito-borne diseases. The data from 2003 to 2010 were used for model calibration, and the rest were used for validation. The PCMP estimates parameters using a simulated annealing method that uses the Markov chain Monte Carlo procedure (see [28]). In parameter optimization procedures, we set the likelihood of the observed data over calibration periods as the objective function. We assumed that the number of observed trapped adult mosquitoes followed a Poisson distribution of which expectation was weekly averaged population obtained from the model for each week. Optimal parameters were determined in the PCMP using this heuristic estimation. The PCMP outputs the captured female population dynamics because the observation data for parameter fitting counted female individuals. We assumed that the capture rate was constant throughout the calculation period and the death of the captured individual did not affect the overall population dynamics. To determine the maximum carrying capacity, *κ*_*max*_, we observed 1% of the active adults captured in the dry-ice traps.

Meteorological data were obtained from the Japan Meteorological Agency database [40], and contained data on air temperature, precipitation, relative humidity, solar radiation, wind speed, and cloud amount that were collected at the meteorological station (35° 41’ 30’’ N, 139° 45’ 00’’ E, 25 m altitude, and 4.4 km from the mosquito observation site). The photoperiod was calculated using the National Oceanic and Atmospheric Administration solar calculator [41].

To predict the mosquito population dynamics under future climatic conditions, we used the climate projections of the Model for Interdisciplinary Research on Climate (MIROC5) [42, 43], which is one of the GCMs. We converted the average daily values of air temperature, relative humidity, solar radiation, cloud amount, and daily cumulative precipitation to the units used for the data collected at the study point, using the inverse distance weighted interpolation method from the original gridded data format. Furthermore, we used the historical scenarios prevalent during 1991–2009 and predicted two RCP emission scenarios for the period 2081–2099, namely, low CO_2_ emissions (RCP 2.6), and high CO_2_ emissions (RCP 8.5) scenarios.

### 2.4 Calculation of habitat environmental conditions

*Aedes albopictus* has five life stages: egg, diapausing egg, larva, pupa, and adult. The first four life stages are aquatic, and the last stage is aerial. As mentioned in Section 2.2, the aquatic environment controls mosquito population via carrying capacity. We calculated the water temperature and soil moisture ratio using the climatic factors in the aforementioned section (Fig 1). The daily water temperature was derived using the method described in [44], which is a simple energy balance model (see [28] for a detailed explanation). According to [37], daily soil moisture in the mosquito habitat was calculated as the difference between water input (precipitation and snowmelt) and output (evapotranspiration). Evapotranspiration was derived using the water balance model [45].

## 3. Results

### 3.1 Validation of PCMP model for *Aedes albopictus*

We applied the PCMP models while considering different rainfall effects and studied the response of carrying capacity to soil moisture (*ν* = 0, 1/5, 1/2, 1). The likelihood during the validation period (2011–2013) and optimized parameters of each model are listed in Table 2.

**Table 2:** Estimated parameter of each model and its fitting to the observations.

Responsiveness of carrying capacity (v)	Log likelihood	*κ*_*max*_	*α*_d1_	*α*_d2_	*α*_t1_	*α*_t2_	*β*_d1_	*β*_d2_	*β*_t1_	*β*_t2_
0	−1047	10 ^1.75^	10 ^4.19^	10 ^2.80^	10 ^0.69^	10 ^2.43^	14.223	11.527	30.000	9.458
1/5	−1061	10 ^1.80^	10 ^4.92^	10 ^3.28^	10 ^0.73^	10 ^2.38^	14.286	11.520	29.743	9.460
1/2	−1089	10 ^1.86^	10 ^5.06^	10 ^4.40^	10 ^4.78^	10 ^2.63^	14.325	11.528	29.058	9.449
1	−1067	10 ^2.00^	10 ^5.10^	10 ^3.84^	10 ^4.70^	10 ^2.42^	14.420	11.518	28.822	9.458

The parameters of diapause were estimated to be: photoperiod of 14.3 h for *β*_d1_ (DOY [day of year] = 200), almost 29 weeks from the first day of the year in Tokyo (see S1 Fig in S1 File) and temperature of approximately 29 °C for *β*_t1_ (maximum value of Ta_week_ on average from 2003 to 2013 was 28.7 °C at 32 weeks). The parameters of diapause-breaking were estimated to be approximately 11.5 h for *β*_d2_ (DOY = 64, between 9 and 10 weeks at Tokyo) and 9.5 °C for *β*_t2_ (almost the same as the *Tw*_week_ on average from 2003 to 2013 at 10 weeks at Tokyo). Additionally, the parameters of the photoperiod sensitivity *α*_d1_ and *α*_d2_ were larger than those of temperature sensitivity *α*_t1_ and *α*_t2_. The models with *v* = 0 and 1/5 were estimated to have a low temperature dependency on diapause, because the temperature sensitivity of diapause *α*_t1_ was smaller than that of *α*_d1_.

**Fig 2:**
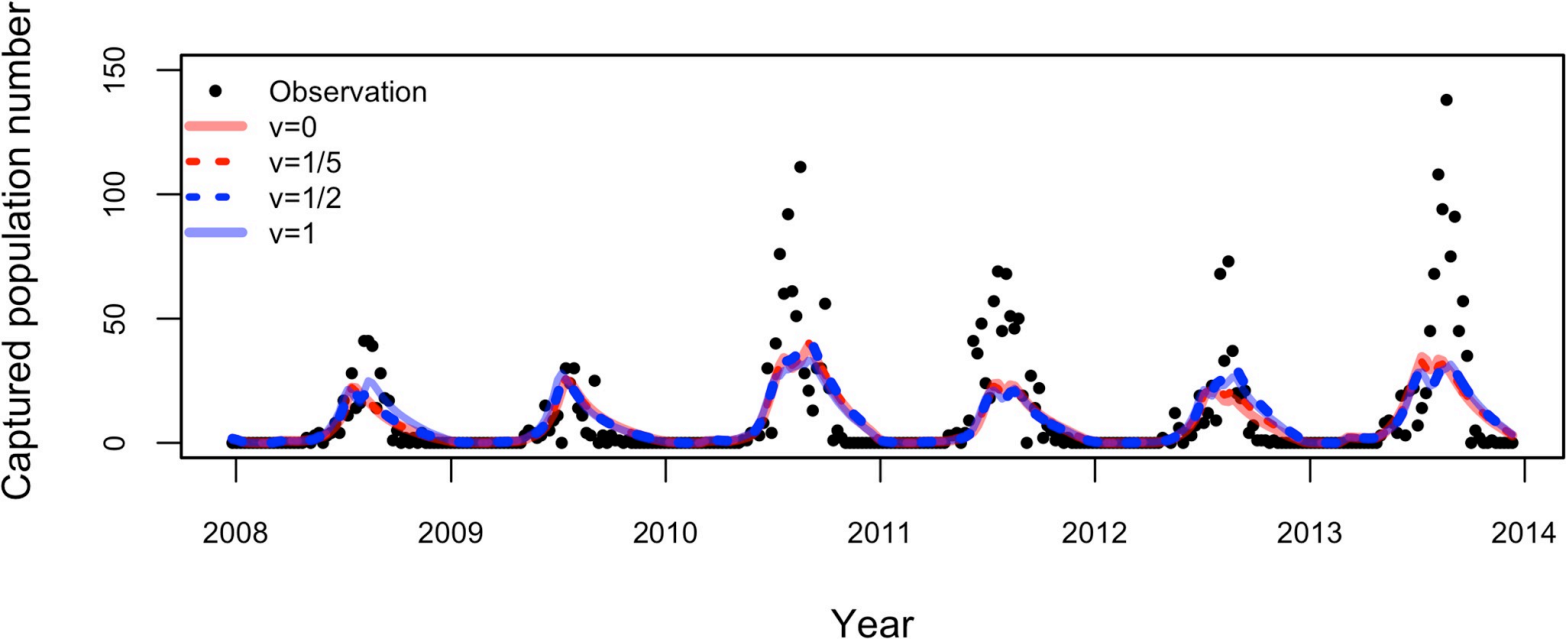
Population dynamics during calibration (2003–2010) and validation (2011–2013) periods. The black circle represents the observation data, and lines represent the predicted population dynamics using different models, respectively.

The predicted population dynamics of all models were almost identical, as shown in Fig 2, although the model with *v* = 0 had the highest prediction accuracy based on log-likelihoods (Table 2). All models have a bias that underestimates during the most active period and overestimates at the end of the active period. Table 3 shows the prediction accuracy of the model for the three characteristic periods, beginning and termination of diapausing, and the most active season, in terms of the log-likelihood per period.

**Table 3:** Mean log-likelihoods of three specific periods during the simulated term of validation.

Responsiveness of carrying capacity (v)	Weeks		
	16–23	29–36	42–49
0	−18.728	−114.826	−42.870
1/5	−18.496	−115.690	−44.011
1/2	−18.458	−116.201	−47.013
1	−17.698	−121.953	−45.835

The model having the responsiveness of carrying capacity value of *v* = 1 had better reproducibility at the termination of the diapausing periods, and the model having the *v* value of 0 had good reproducibility in the most active season, compared to the other models. The intermediate values of the response function of carrying capacity to soil moisture in cases where *v* = 1/2 and 1/5, larger log-likelihoods were observed than the cases wherein *v* = 0, which in turn, had larger log-likelihoods than the proportional response case where *v* = 1. As shown in Table 3, the models with *v* = 1/5 and *v* = 0 indicated a similar pattern: the log-likelihood was lower at the beginning of diapause and higher during the active period. Notably, the non-linear response of carrying capacity to soil moisture enhanced the mosquito population size, even if precipitation was low and reached the plateau quickly in the responding as well as the fixed case (*v* = 0), because the function is highly responsive to soil moisture. This was not true when the responsiveness was low, as shown in the case of *v* = 1/2, and the pattern in the log-likelihood of the model having value of *v* = 1/2 was different from that having values of *v* = 0 and 1/5.

### 3.2 Understanding output bias from input meteorological, observed, and historical data using global climate model

**Fig 3:**
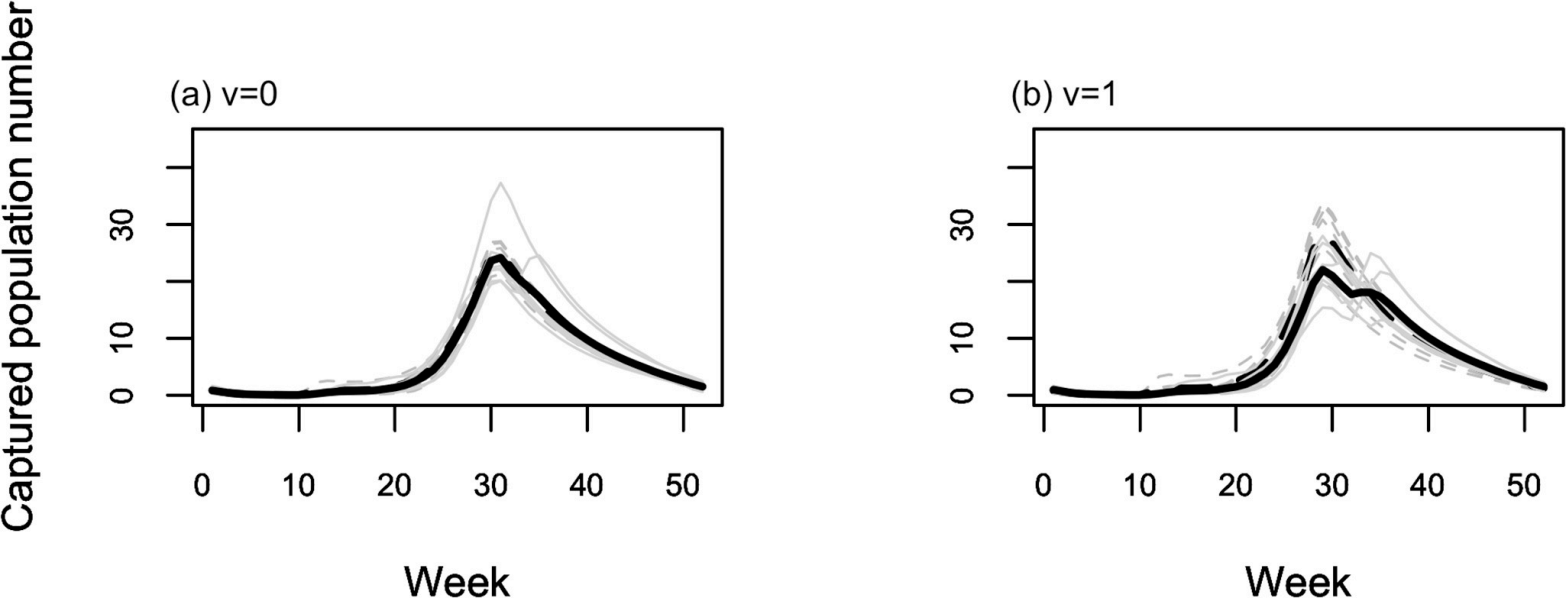
Population dynamics (2003–2009) with two different climate data using model having (a) *v* = 0 and (b) *v* = 1. Solid line represents the prediction using the observed climate data and dashed line represents those with global climate model (GCM) historical data. Light and strong colors indicate the result of each year and its average, respectively.

**Fig 4:**
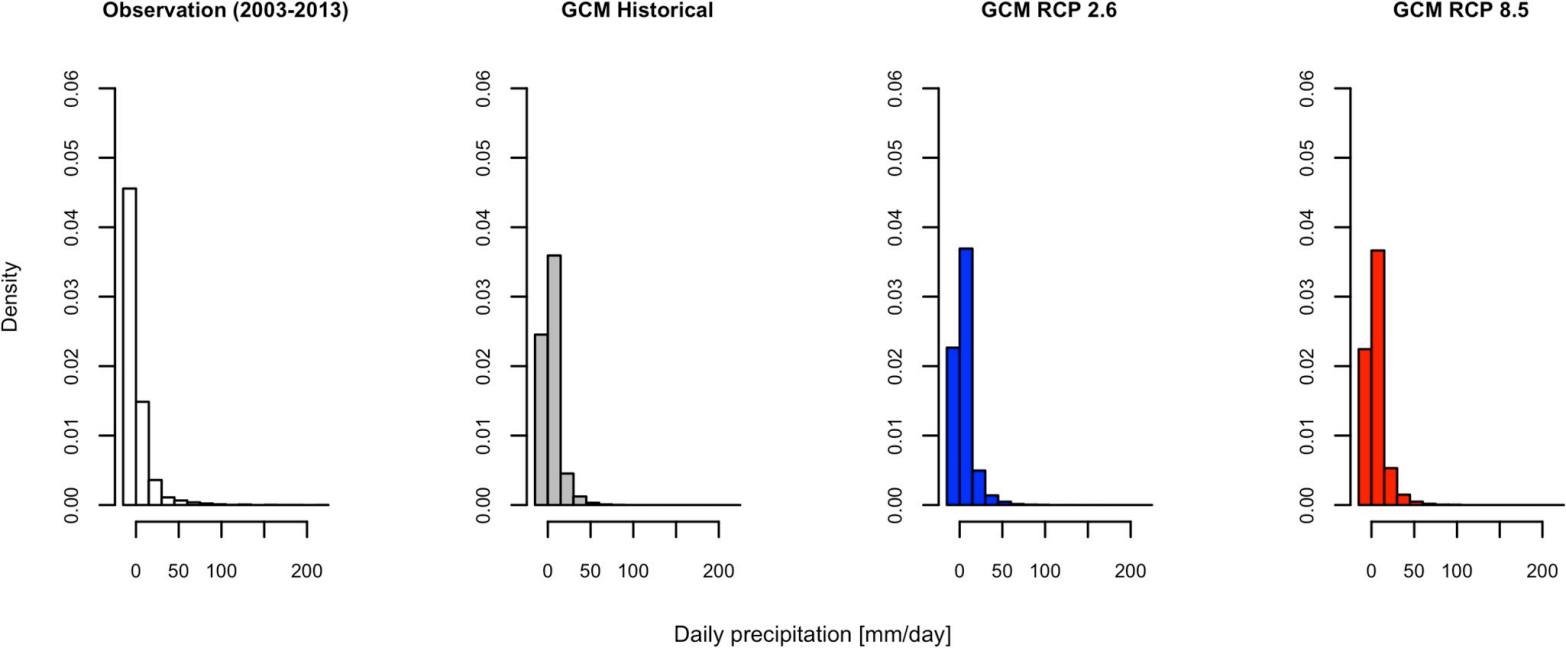
Differences in the precipitation among observed and MIROC (a global climate model) data. The leftmost bar in each histogram represents the frequency of zero precipitation.

**Fig 5:**
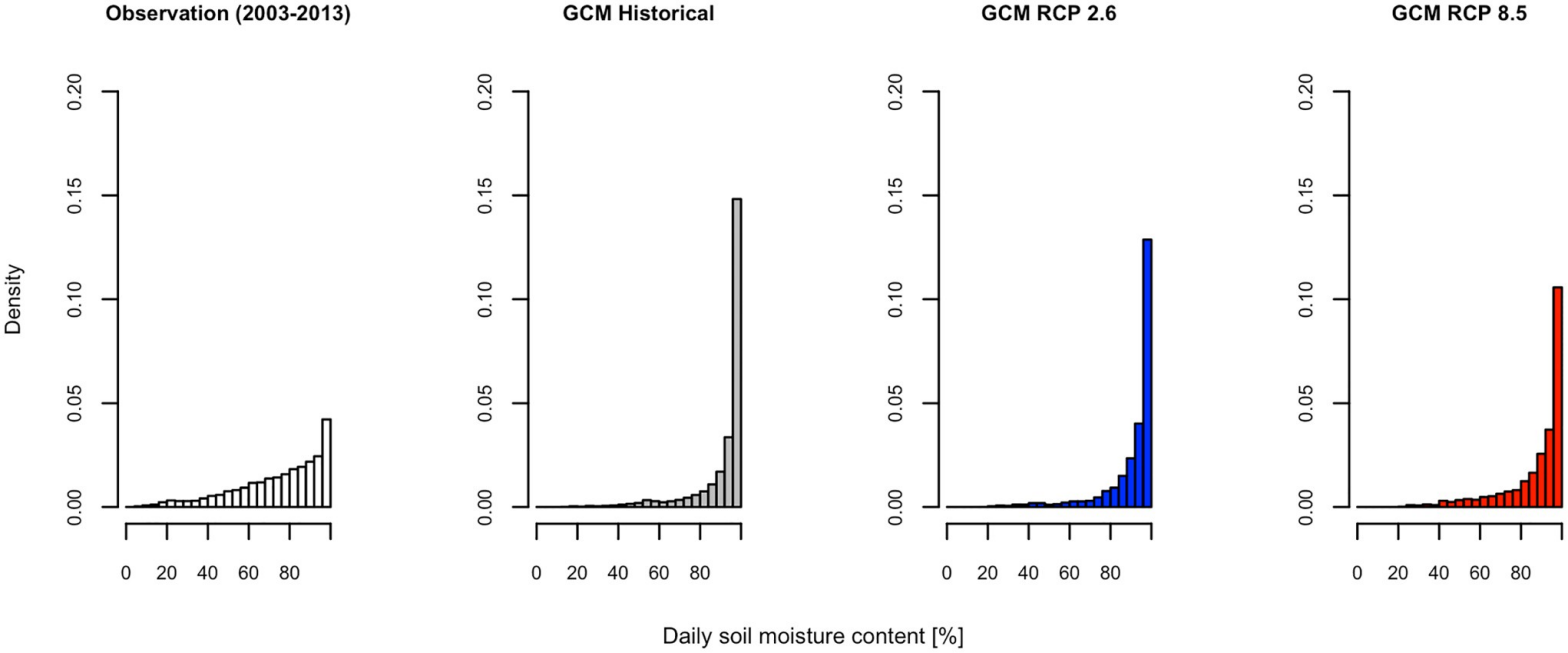
Differences in the soil moisture content among observed and MIROC (a global climate model) data.

In this study, the prediction of mosquito populations was made using the PCMP model. The mosquito populations during the historical (1991–2009) and future (2081–2099) periods were simulated using the PCMP model coupled with a GCM model (MIROC5). First, we showed two different predicted population dynamics for *Ae*. *albopictus* using the models having responsiveness values of *v* = 0 and 1 during 2003–2009: one derived by coupling with the observed climate data, and the other with the GCM model. As shown in Fig 3, the active periods of *Ae*. *albopictus* in the predicted population dynamics were almost the same between the two predictions if the model having a value of *v* = 0 was used (Fig 3a), whereas the population peak in the historical simulation was larger than that obtained using observed meteorological data for *v* = 1 (Fig 3b). In the cases where *v* = 1, the peak population (on average) estimated using the GCM historical data was 1.29 times larger than that estimated using observed meteorological data, whereas the bias was 1.03 in the case of *v* = 0 (see S1 Text in S1 File for calculating these biases). The bias arises from the fact that the precipitation pattern in GCM differed from the observed data, as shown in Fig 4, which shows the frequencies of precipitation in the observation, GCM historical, and GCM future climate (see S2 Text in S1 File for the output procedure of Fig 4). The frequency of non-precipitation days was comparatively higher in the observed data, while the frequency of a small amount of precipitation was higher in the GCM data (see S1 Table in S1 File). Additionally, the GCM data had no measurements for a large amount of precipitation. These differences in precipitation patterns between the observed and GCM data caused a large discrepancy in the frequency of soil moisture content, as shown in Fig 5, which regulates the carrying capacity and appears as a difference in peak population. Assuming that there would be a bias in the peak population in the GCM data, we made predictions of mosquito population dynamics under future climate conditions.

### 3.3 Predictions of mosquito population with and without the incorporation of rainfall effect on carrying capacity

**Fig 6:**
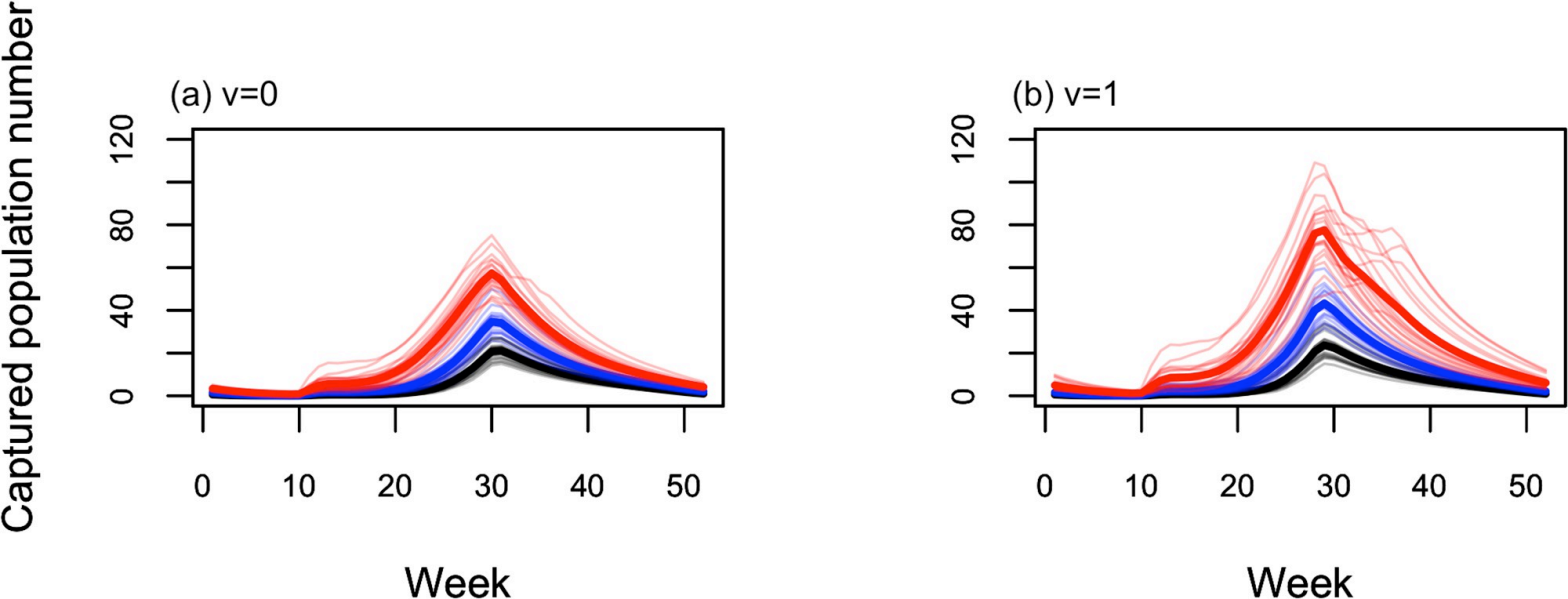
Different patterns of population dynamics in the future between two models having different responsiveness values: (a) *v* = 0 and (b) *v* = 1. Projected population dynamics with global climate model (GCM) data (historical, RCP 2.6, and RCP 8.5). Lines with light colors represent the population dynamics of each year and lines with strong colors represent the averaged population dynamics, and black, blue, and red lines represent the predicted population dynamics using historical data (1991–2009), the projected population dynamics with GCM data of RCP 2.6 scenario (2081–2099), and that with GCM data of RCP 8.5 scenario (2081–2099), respectively.

Fig 6 shows the simulation results of the population dynamics under future climate conditions for each CO_2_ emission scenario. In our study, the population increased in the spring season and the termination of active period after autumn was prolonged in the simulated result under future climate conditions as CO_2_ emissions increased. In particular, the termination timing was prolonged even though the beginning of the mosquito activation period did not change in either case (*v* = 0 and *v* = 1). The peak in the number of individuals was fixed at the 30^th^ week and did not change depending on the emission scenarios and year of simulation, regardless of the models (Table 4). Conversely, in RCP 8.5 (the scenario with the highest CO_2_ emission), there was a large fluctuation in the population number in the simulated years; in the historical simulation and the RCP-2.6 simulation, there were only small fluctuations. Abrupt increases in population immediately after diapause-breaking were found in the RCP 8.5 model, while populations tended to gradually increase in the RCP 2.6 and historical simulations.

**Table 4:** Peak population number on averaged dynamics with Global Climate Model (GCM) and the peak week.

	GCM Scenario	*v* = 0		*v* = 1	
Averaged population number [week number at peak]	Historical	21.1	[31]	23.7	[29]
	RCP 2.6	34.7	[30]	43.3	[29]
	RCP 8.5	57.4	[30]	77.6	[29]

In this study, we compared the simulated populations using models under different future climate conditions (*v* = 0, fixing the carrying capacity, and *v* = 1, incorporating the effect of rainfall on carrying capacity), while focusing on how precipitation affects the prediction of mosquito population dynamics. The population number and fluctuations in the population number in the simulated years predicted by the model having a responsiveness value of *v* = 0 were smaller than those predicted by the model having a responsiveness value of *v* = 1 throughout the simulation period. Peak population number simulated with RCP 8.5 predicted by the model having a responsiveness value of *v* = 1 was 1.35 times (1.09 times if bias is corrected) larger than the predictions obtained by the model having a responsiveness value of *v* = 0 (Table 4).

Compared with the case where the non-linear response in the carrying capacity was considered (S2 Fig, S3 Text in S1 File), a larger population number was simulated because the estimated maximum carrying capacity *κ*_max_ of the model having a responsiveness value of *v* = 1 was greater than that estimated using models of other values (Table 2). In addition, soil moisture content of 100% provided the maximum carrying capacity, as shown in Fig 5.

## 4. Discussion

Through the simulation of population dynamics of the Asian tiger mosquito (*Ae*. *albopictus*), a vector of dengue virus, we could deduce that climate change may increase the potential risk of dengue fever outbreak in the future. The PCMP model reproduced the seasonal trends of mosquito population activity, which was observed weekly at the study point from 2003 to 2013 [i.e., under current temperate-climate conditions (Table 2)]. In particular, the predictions of both the beginning of the active season for mosquito and the peak of the active period are important for warning of vector-borne diseases. The reproducibility of mosquito population dynamics at the termination of diapause was more accurate and effective when the model incorporated rainfall as one of its parameters, whereas the estimated environmental cues for diapause-break were almost identical in both mosquito population models that incorporated and overlooked the effect of rainfall. Furthermore, the larger population, simulated by a model that incorporated the effects of rainfall at the beginning of the active season, was estimated more accurately than that of the population simulated by the model that did not consider the rainfall effect in future climate conditions. This study claims that the risk assessment for future dengue fever outbreak requires precipitation data, whereas the current risk assessment is based on temperature (mainly for the estimation of habitat distribution and population number of mosquitoes) [2].

On the other hand, the output of the PCMP model underestimates population peaks and overestimates them at the end of the active season under current climate conditions. One reason for these inaccuracies is the use of single point observation data for parameter estimation, which results in biased estimation. Using multilocation data could have solved this problem; however, it was not possible to obtain time-series data on the number of mosquitoes around Tokyo and even throughout Japan except for open data available in [39]. Applying the PCMP model to multilocation data is an issue that must be addressed in the future. Another reason is that observation errors caused by mosquito behaviors and detailed adult behavior were not considered in the model. In this study, for simplicity, the PCMP model assumed that the mosquito capture rate was 1%, whereas the mosquito capture rate varied under natural conditions, depending on the habitat’s meteorological conditions. Therefore, the observation error should be examined to reveal the range of uncertainty needed by using outdoor trapping, because the captured population might change considerably. Moreover, the PCMP did not consider the physical activity of the mosquitoes. Accordingly, the model could not consider unmoving individuals that were inactivated by the cool temperature of the habitat in autumn and early winter. Furthermore, the mortality rate will be higher in field conditions [46] than that assumed in this model, which is based on observations in the laboratory [24]; hence, the PCMP model may overestimate the mosquito populations in autumn. The model underestimates the mosquito populations in summer as a result of selecting a large likelihood values during summer and autumn seasons. As the number of observation data is small, the variance of Poisson distribution is small, and as a result, the likelihood has a larger value if the difference between observation and prediction by the model is small.

This study demonstrates the significant differences in the projection of mosquito populations even if the models that consider the effects of rainfall can predict the current population dynamics (Fig 6). There was no significant difference in the reproduction accuracy between the two models (one with constant carrying capacity and the model with a variable carrying capacity proportional to soil moisture content). This was the case throughout the simulated period for the current temperate-climate conditions (Table 2), but large differences were observed in future projections. Larger populations and their fluctuations during the active season were simulated in the future projection as the responsiveness of carrying capacity (to soil moisture) increased (Fig 6 and S3 Text, S3 Table in S1 File). One reason of this difference is the different size of carrying capacity (*κ*_max_ in Table 2). A larger carrying capacity for the larval stage was estimated as parameter *v* in the model assumes a larger value. Another reason was the rainfall pattern of the GCM. Incorporating the effect of rainfall into the model or overlooking this effect caused a different level of bias on the simulated mosquito population between the output obtained using the observed meteorological data and that using GCM data, as shown in Fig 3. The frequency of occurrence of days using high soil moisture obtained from GCM meteorological data was higher than that obtained from observed meteorological data, as shown in Figs 4 and 5. To project the population of *Ae*. *albopictus* in the future, it is important to consider this effect because of the extreme amount of rainfall forecasted in temperate regions [13]. Notably, the GCM could not generate the daily rainfall pattern, even though it could reflect the rainfall pattern on a monthly scale. It is necessary to assess mosquito-borne diseases to improve the generation of rainfall patterns on a daily scale. In addition, the generated pattern of future climate differs depending on GCMs. We also made projections using climate data of Geophysical Fluid Dynamics Laboratory (GFDL) and Hadley Centre Global Environment Model [43], which are GCMs other than MIROC to confirm whether the GCM output pattern might make a difference in the projection of population dynamics under future climatic conditions (See S4 Text, S3 Fig, and S4 Table in S1 File). The difference between RCP 2.6 and RCP 8.5 in the output of PCMP model was consistent with that derived using the climate data of MIROC. It was confirmed that the assumption of the responsiveness of carrying capacity to soil moisture (*v*) will cause a difference in mosquito population during active season in the future.

Different patterns of mosquito population dynamics were predicted (for the future) by each model at the beginning of the active season of mosquitoes, indicating that different approaches must be prepared for the risk management of dengue fever to prevent an outbreak. This kind of variation in projection always occurs if the model incorporates environmental factors that control population dynamics; notably, the pattern of these factors may be altered in the future. Therefore, the differences in assumptions of the models can result in significant differences in future projections if there are multiple models that can reproduce the current observation data effectively. For optimal risk assessment, it is necessary to perform a comparison among these models. The inter-model comparison method has already been carried out using the future projection from climate models, such as the Coupled Model Inter-comparison Project [13] and crop models for agriculture such as the Agricultural Model Inter-comparison and Improvement Project [47]. The improved PCMP model in this study incorporates several meteorological factors that affect the output. The mechanistic population model with diapause (MPAD) [24, 48] and the models used by Tran et al. [22] are climate-driven models of mosquito population dynamics for *Ae*. *albopictus*. For the risk assessment of dengue outbreaks, mosquito population models for *Ae*. *aegypti* (e.g., the dynamic mosquito simulation model) [49] and matrix population model [23], also need to be implemented, and these results must be compared with each other.

The PCMP applied in our study estimates the photoperiod and temperature sensitivity parameters related to the diapause to fit the observations obtained through the study in Tokyo such that the prediction and projection of the population dynamics are tailored to the area. Prediction of population dynamics in *Ae*. *albopictus* was conducted by Jia et al. in Guangzhou, China [48], in which the temperature in the MPAD model [24] was assumed to add 1, 3, and 5 °C constantly to the current climatic condition during specific seasons. The condition of diapause was fixed as *T*_ave_ < 21 (°C) and *D*_ave_ < 13.5 (h) for diapause and *T*_ave_ > 10.5 (°C) and *D*_ave_ > 10.25 (h) for diapause breaking, in which *T*_ave_ and *D*_ave_ refer to the weekly average of daily temperature and daily photoperiod, respectively, based on the former experimental findings (for diapausing [50]: in Shanghai [51]; in Nagasaki; for awaking [52]: in Rome). The diapause in the MPAD model was assumed to have been switched on and off. These critical temperatures or photoperiods for diapause determine the appearance and disappearance timing of *Ae*. *albopictus*.

*T*_ave_ < 21 (°C) and *D*_ave_ < 13.5 (h) in Tokyo were realized during weeks 39–40 and 33–34 from the first day of the year from 2003 to 2013, and *T*_ave_ > 10.5 (°C) and *D*_ave_ > 10.25 (h) were realized approximately at 12 weeks and during weeks 5–6. The first appearance in a year and the last appearance, as well as the peak timing, will be later than the observation if the model used those thresholds.

Thus, despite the fact that the observation data were limited to the adult population, the PCMP model reproduces the weekly observation effectively. This suggests that the parameter estimation for each habitat was effective for the construction of the population dynamics model to fit observed data at the observation site, and also for forecasting future population dynamics at the target site. However, the mosquito’s adaption abilities imply that it can adapt to the environment under climate change conditions. Therefore, we applied the optimized parameters to the current observations to predict future population dynamics and ignored the possible changes in these parameters in this study. Notably, the physiological traits of the mosquitoes may evolve through generations, leading to alterations in population responses to climate change in the far-future. Therefore, it will be necessary to consider the adapted life cycle of mosquitoes for forecasting population dynamics accurately and assessing the outbreak of dengue fever under climate change conditions. Incidentally, projections of population dynamics by the PCMP were made for the near future (2031–2049), when traits are unlikely to change much (see S5 Text, S4 Fig, and S5 Table in S1 File for details). The differences in patterns of population dynamics derived from the responsiveness of carrying capacity to soil moisture (*v*) and the differences in the output of the PCMP model between the two RCPs were consistent with those of the distant projection for 2081–2099.

## Supporting information

S1 File (DOCX)
