# Supplementary material for: Modeling the effect of rainfall changes to predict population dynamics of the Asian tiger mosquito Aedes albopictus under future climate conditions
Source: PLoS One. 2022 May 25;17(5):e0268211. doi: 10.1371/journal.pone.0268211 (PMC9132271; doi:10.1371/journal.pone.0268211)
Supplement: S1 File — (DOCX) [file pone.0268211.s001.docx]

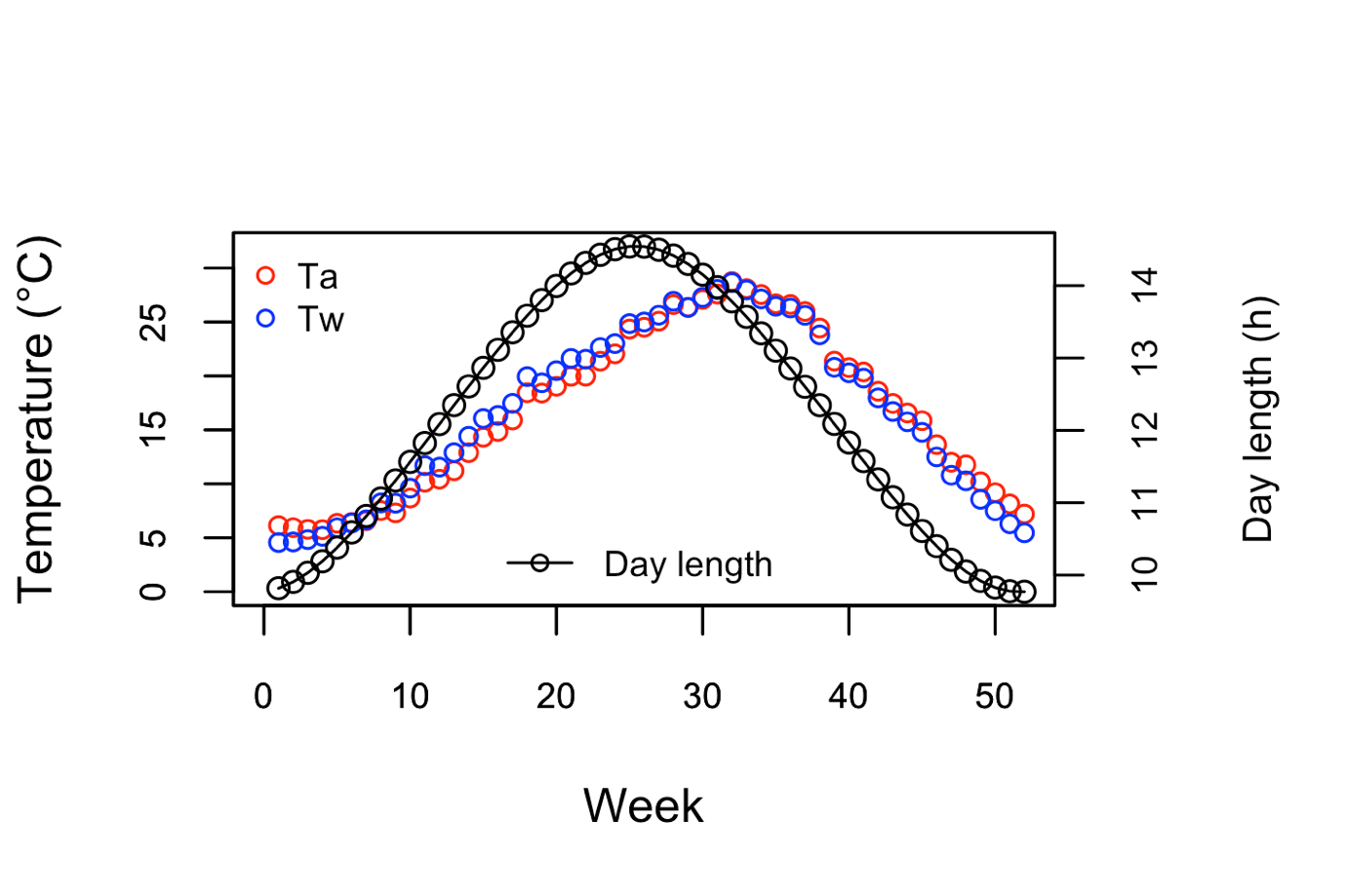


**S1 Fig.** Weekly meteorological variables on average from 2003 to 2013 measured at Tokyo


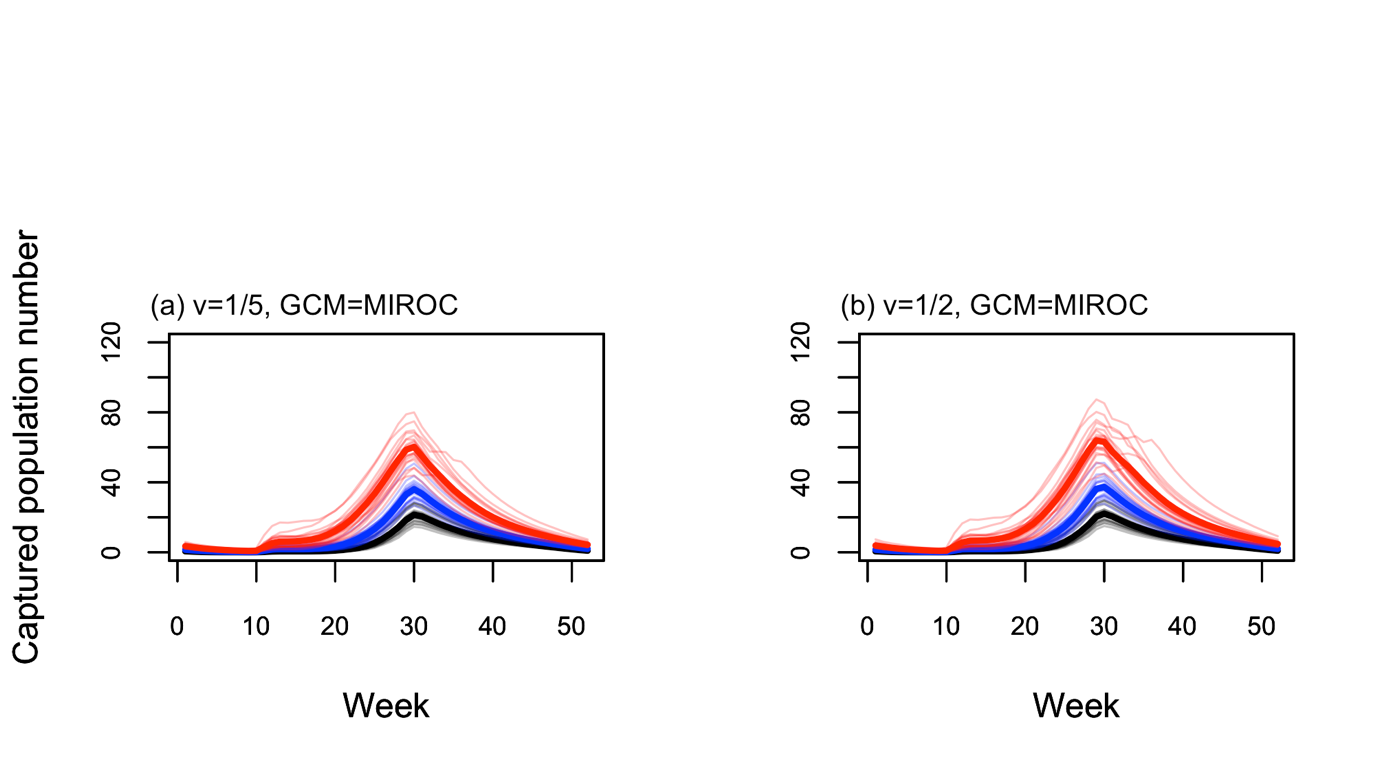


**S2 Fig.** Projected population dynamics with the climate data of MIROC (Historical, RCP 2.6, and RCP 8.5), a global climate model (GCM). Lines with light colors represent the population dynamics of each year and lines with strong colors represent the averaged population dynamics; black, blue, and red lines represent the predicted population dynamics with historical data (1991–2009), projected population dynamics with MIROC data of RCP 2.6 scenario (2081–2099), and that with MIROC data of RCP 8.5 scenario (2081–2099), respectively


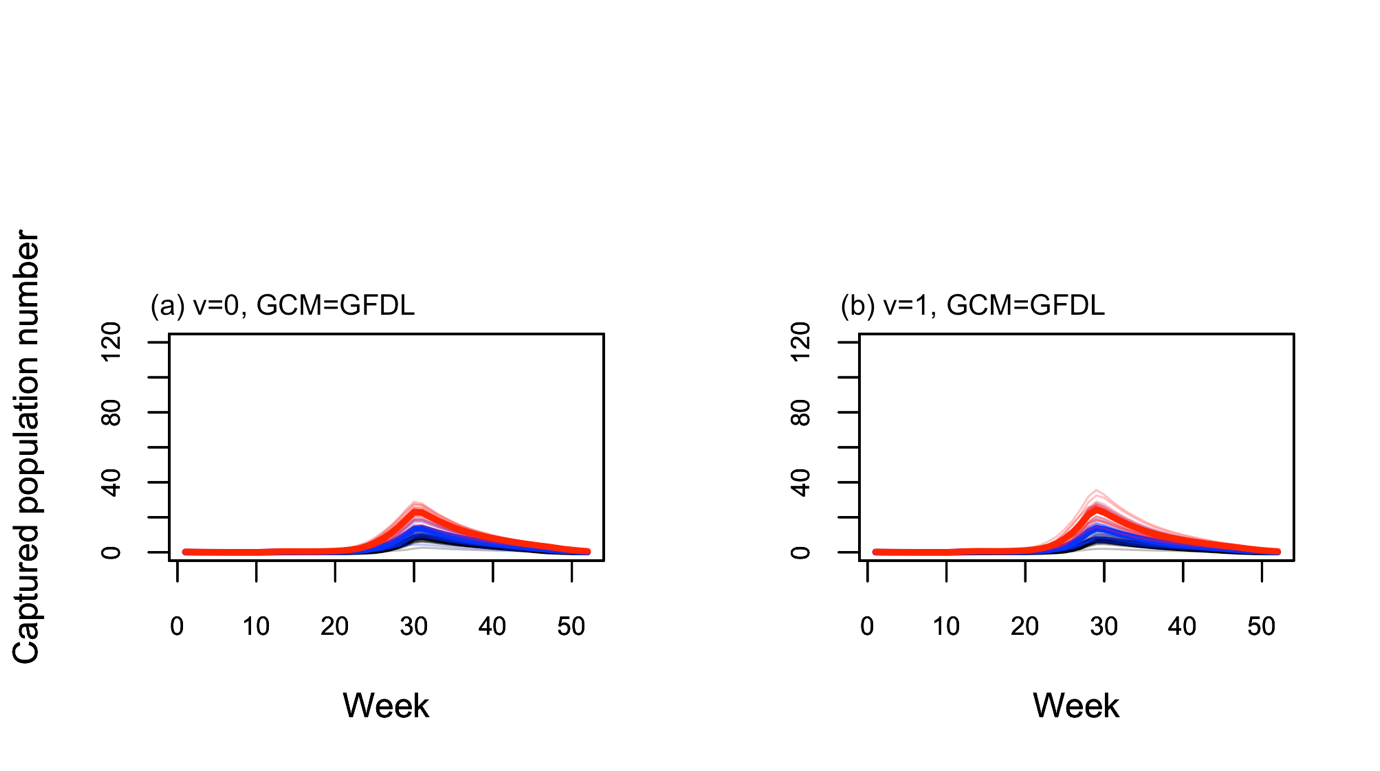

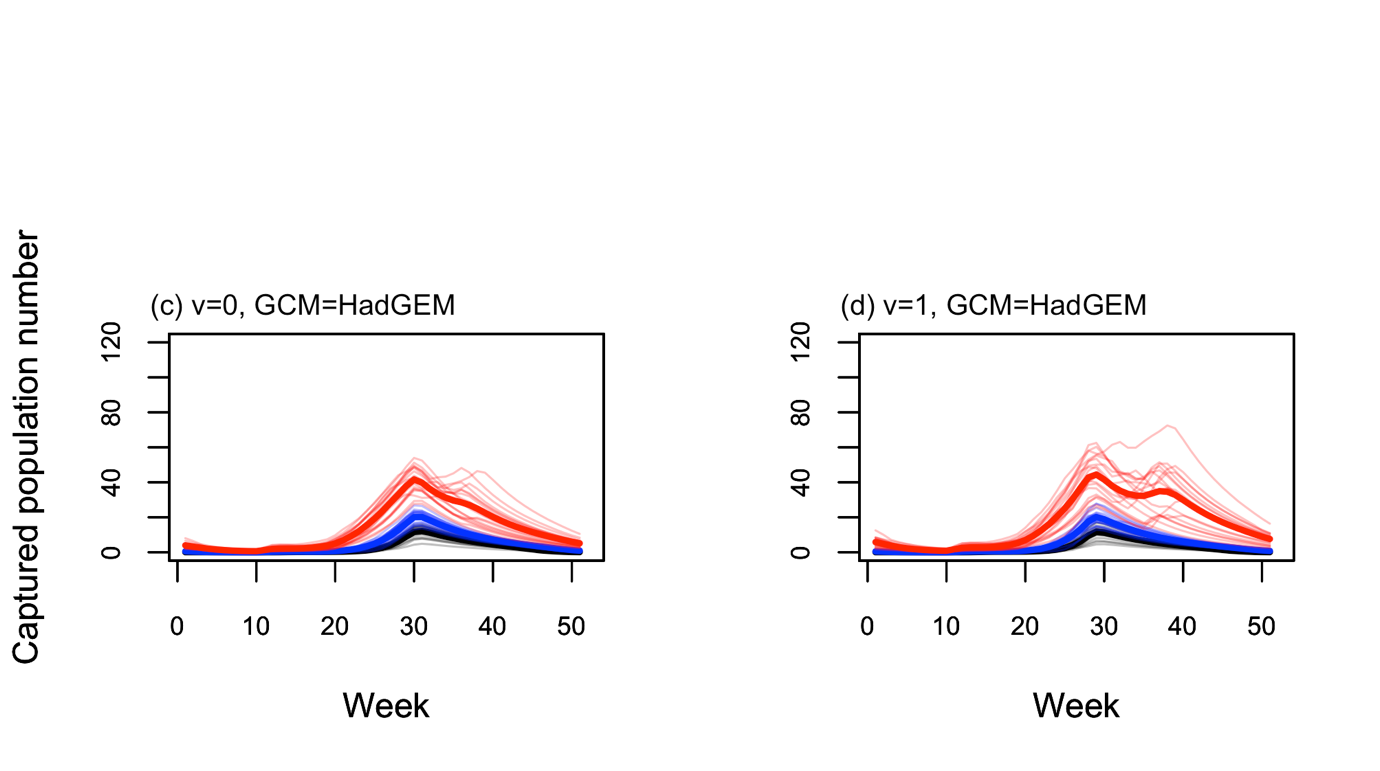


**S3 Fig.** Projected population dynamics with the climate data of GFDL and HadGEM (Historical, RCP 2.6, and RCP 8.5), global climate models. Upper panels represent the result using GFDL and lower panels using HadGEM. Left-sided panels represent the results of output of PCMP model with *v* = 0, and right-sided panels shows those with *v* = 1. Lines with light colors represent the population dynamics of each year and lines with strong colors represent the averaged population dynamics; black, blue, and red lines represent the predicted population dynamics with historical data (GFDL;1987–2005, HadGEM; 1986–2004), projected population dynamics with GCM data of RCP 2.6 scenario during 2082–2100 for GFDL and 2080–2098 for HadGEM, and that with GCM data of RCP 8.5 scenario, respectively


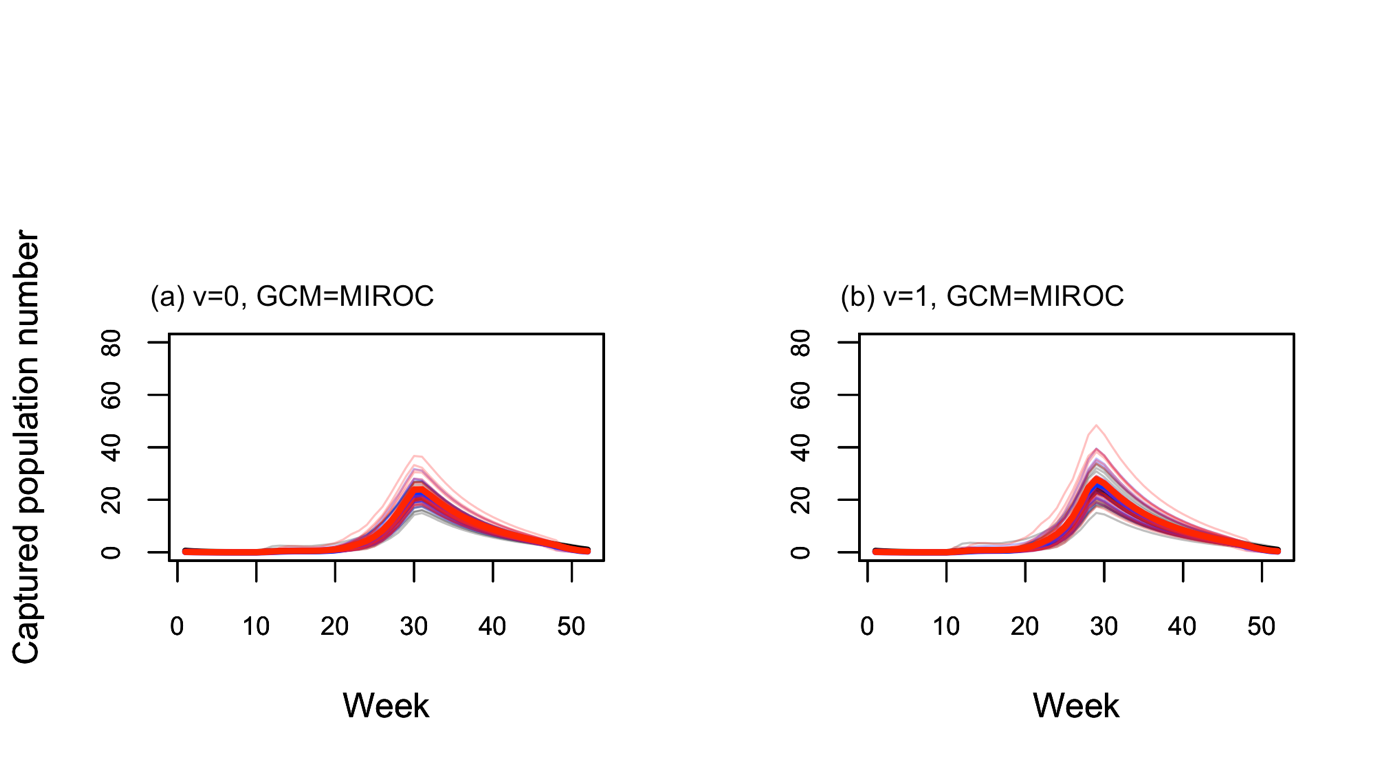


**S4 Fig.** Projected population dynamics in near-future with the climate data of MIROC (Historical, RCP 2.6, and RCP 8.5). Lines with light colors represent the population dynamics of each year and lines with strong colors represent the averaged population dynamics; black, blue, and red lines represent the predicted population dynamics with historical data (1991–2009), projected population dynamics with GCM data of RCP 2.6 scenario (2031–2049), and that with GCM data of RCP 8.5 scenario (2031–2049), respectively

S1 Table. Frequency of zero precipitation

| Reference data | Period | Relative frequency of zero precipitation |
| --- | --- | --- |
| Observation | 2003–2013 | 0.684 |
| MIROC/Historical | 1991–2009 | 0.368 |
| MIROC/RCP 2.6 | 2081–2099 | 0.340 |
| MIROC/RCP 8.5 | 2081–2099 | 0.337 |

**S2 Table.** Peak population number on the averaged dynamics with the climate data of MIROC and the peak week

|  | GCM / Scenario | *v* = 1/5 | | *v* = 1/2 | |  |
| --- | --- | --- | --- | --- | --- | --- |
| Averaged population number [week number at peak] | MIROC/Historical | 21.45 | [30] | 22.0 | [30] | |
|  | MIROC/RCP 2.6 | 36.01 | [30] | 37.4 | [30] | |
|  | MIROC/RCP 8.5 | 60.24 | [30] | 63.9 | [29] | |

S3 Table. Coefficient of variance at peak timing during simulated period using MIROC data

|  | GCM / Scenario | *v* = 0 | *v* = 1/5 | *v* = 1/2 | *v* = 1 |
| --- | --- | --- | --- | --- | --- |
| Coefficient of variance | MIROC/Historical | 0.183 | 0.197 | 0.205 | 0.241 |
|  | MIROC/RCP 2.6 | 0.161 | 0.155 | 0.150 | 0.164 |
|  | MIROC/RCP 8.5 | 0.145 | 0.154 | 0.173 | 0.196 |

**S4 Table.** Peak population number on the averaged dynamics with the climate data of GFDL during 2082–2100 and HadGEM during 2080–2098 and the peak week

|  | GCM / Scenario | *v* = 0 | | *v* = 1/5 | | *v* = 1/2 | | *v* = 1 | |
| --- | --- | --- | --- | --- | --- | --- | --- | --- | --- |
| Averaged population number [week number at peak] | GFDL/Historical | 8.9 | [31] | 8.2 | [30] | 8.0 | [30] | 7.2 | [29] |
|  | GFDL/RCP 2.6 | 13.7 | [31] | 13.5 | [30] | 13.6 | [30] | 13.7 | [29] |
|  | GFLD/RCP 8.5 | 23.0 | [30] | 23.4 | [30] | 23.5 | [30] | 24.3 | [29] |
|  | HadGEM/Historical | 12.1 | [31] | 11.8 | [30] | 11.9 | [30] | 11.7 | [29] |
|  | HadGEM/RCP 2.6 | 20.1 | [31] | 20.1 | [30] | 19.9 | [30] | 20.1 | [29] |
|  | HadGEM/RCP 8.5 | 41.6 | [30] | 41.5 | [30] | 40.7 | [30] | 44.4 | [29] |

**S5 Table.** Peak population number in the near-future on averaged dynamics with MIROC data during 2031–2049 and the peak week

|  | GCM/Scenario | *v* = 0 | | *v* = 1/5 | | *v* = 1/2 | | *v* = 1 | |
| --- | --- | --- | --- | --- | --- | --- | --- | --- | --- |
| Averaged population number [week number at peak] | MIROC/Historical | 21.1 | [31] | 21.5 | [30] | 22.0 | [30] | 23.7 | [29] |
|  | MIROC/RCP 2.6 | 22.3 | [31] | 23.1 | [30] | 24.1 | [30] | 26.3 | [29] |
|  | MIROC/RCP 8.5 | 24.0 | [31] | 24.8 | [30] | 25.7 | [30] | 27.9 | [29] |

S1 Text. Bias correction of mosquito population dynamics derived using the global climate model (GCM) meteorological data

The physiology-based climate-driven mosquito population (PCMP) model requires daily meteorological data and the pattern of mosquito population dynamics, because the model output depends largely on the input meteorological data. MIROC5 [42, 43], a GCM, has a biased precipitation pattern; therefore, the mosquito population pattern (as the output) of the PCMP differs from that obtained using the observed meteorological data (OBS). In this study, we corrected this bias by calculating the difference between the weekly averaged population numbers from 2003 to 2009 using the GCM and OBS and multiplying them weekly, because both GCM and OBS meteorological data overlapped during this period.

S2 Text. Summarizing procedure of soil moisture content for the determination of habitat conditions

The soil moisture content determines the carrying capacity of the PCMP models. We derived the soil moisture content by using the water balance model for soil moisture [45] along with a micrometeorology model [37]. The variables were derived on a daily time scale. To summarize the distribution during the calculation period of mosquito population dynamics, histograms of the environmental variables, including daily precipitation, were created. For precipitation, the observation was set as zero if the daily accumulated amount was lower than 0.5 mm. To match the conditions, we set the GCM output to zero if it was less than 0.5. To distinguish between 0 and the others for daily precipitation, we converted 0 values to −5 to appear at the left most bar by counting 0 in the negative range in histograms. All histograms was constructed using R^©^ ver.4.0.2.

S3 Text. Projecting population dynamics using the physiology-based climate-driven mosquito population (PCMP) model and non-linear response to soil moisture (*v* = 1/5 and 1/2)

Where the model assumed a non-linear response of carrying capacity to soil moisture (*v* = 1/5 and 1/2), population numbers at the peak time assumes an intermediate value between those in the model with *v* = 0 and 1.

S4 Text. Predictions and projections of population dynamics by the PCMP model using two different sets of GCM data

Predictions and projections of population dynamics were made using the climate data of GFDL and HadGEM, global climate models [43]. Predictions of population dynamics with GFDL historical data (1987–2005) and with HadGEM historical data (1986–2004) were confirmed. Projections during 2082–2100 for GFDL and during 2080-2098 for HadGEM were also confirmed. Regarding the period of climate data on both GFDL and HadGEM, it was not possible to obtain data for the same period as provided by MIROC data. Note that the climate data provided by HadGEM is 360 days a year. The biases of output of PCMP with historical data from that with observed meteorological data were derived as shown in Fig 3 in the main text (GFDL; 2003–2005, HadGEM; 2003–2004). Using GFDL historical data, the estimated peak population (on average) was 0.42 times smaller than that estimated using observed meteorological data in the case of *v* = 1, whereas the bias was 0.44 in the case of *v* = 0. Using HadGEM historical data, the bias was 0.41 in the case of v = 1, and it was 0.30 in the case of *v* = 0. In both cases, the biases were larger than those derived from the output using the MIROC data. This suggests that the MIROC model can reproduce the observed meteorological data well compared to other GCMs. Results of the projections show that the difference in the output of the PCMP model between RCP 2.6 and 8.5 was consistent with that derived using MIROC data. It was confirmed that the responsiveness of carrying capacity to soil moisture (*v*) results in a difference in mosquito population during active season under future climate conditions if the CO_2_ emission is high (RCP 8.5).

S5 Text. Forecasting population dynamics using the PCMP model for the near-future using MIROC data for 2031–2049

Projections of population dynamics in the near-future were made using climate data of MIROC during 2031–2049. As shown in Figure S4, the patterns of population dynamics among three different input climate data, historical (1991–2009), RCP 2.6 (2031–2049), and RCP 8.5 (2031–2049) were almost the same regardless of the responsiveness of carrying capacity to soil moisture (*v*) because climate data among them do not vary much. The projected population size derived using near-future MIROC RCP 8.5 data during 2031–2049 was the largest compared to others, and the prediction for the historical during 1991–2009 was the smallest, as shown in Table S5. This pattern is consistent with those obtained for the distant future (2081–2099) presented in the main text. It is also identical to the result of the distant projection wherein the population number predicted by the model having a responsiveness value of *v* = 0 was smaller than that predicted by the model having a responsiveness value of *v* = 1, throughout the simulated future period. However, the projected population sizes at the peak week in the near-future were smaller than those in the far-future. Because temperature, one of the major driving forces of the PCMP model, in the near-future climate conditions assumes an intermediate value between those under historical and far-future conditions.
