# Supplementary material for: Modeling the effect of rainfall changes to predict population dynamics of the Asian tiger mosquito Aedes albopictus under future climate conditions
Source: PLoS One. 2022 May 25;17(5):e0268211. doi: 10.1371/journal.pone.0268211 (PMC9132271; doi:10.1371/journal.pone.0268211)
Supplement: Attachment — Submitted filename: Reviwer Comment PONE-D-21-35458.docx [file pone.0268211.s002.docx]

**PONE-D-21-35458**

**Reviewer’s comment**

1. Only one station data is used to develop the equations. As the insect capture rate is only 1% and depends on habitat meteorological conditions, model developed using one station/location may give a biased output.

2. The result is, estimated population is over-estimated in autumn and early winter and under-estimated in summer, as the number of observational points is less.

3. To predict the population dynamic in future, only one GCM has been used. The projection may be biased for a particular GCM? Why not the authors took multiple GCMs?

4. Isn’t it too early to predict for 2081-2099 as the physiological traits and adapted life cycle of the mosquito will change. What about the near-future/ mid-future projections?

I have not to say much on the insect modelling part, but by choosing only one location and one GCM for projection (2081-2099), the validity of the research may be doubtful. I would like to hear the response from the author, therefore recommend for a major change.
