# Supplementary material for: Modeling the effect of rainfall changes to predict population dynamics of the Asian tiger mosquito Aedes albopictus under future climate conditions
Source: PLoS One. 2022 May 25;17(5):e0268211. doi: 10.1371/journal.pone.0268211 (PMC9132271; doi:10.1371/journal.pone.0268211)
Supplement: Attachment — Submitted filename: Mosquitoes-Jan 25-2022.docx [file pone.0268211.s003.docx]

Title: Modeling the effect of rainfall changes to predict population dynamics of the Asian tiger *mosquitoAedes* albopictus under future climate conditions

by Shunji OHTA et al.

The authors fitted the physiology-based, climate-driven mosquito population (PCMP) model for temperate mosquitoes to incorporate egg diapauses for hibernating. They also investigated how incorporating the effect of rainfall on larval carrying capacity (into a model) changes the population dynamics of this species under future climate conditions. A comparison was made with rainfall in the model and without rainfall in the model.

This is a well-written paper that addresses an important topic, the population dynamics of mosquitoes in temperate regions. The authors discussed the article in a consistent manner, and it was a pleasure to read. I recommend acceptance of the article for publication in the journal.

**Minor revision**:

A brief description of how the MCMC simulation method was used to estimate PCMP model parameters would clarify calculations.
