# Supplementary material for: Modeling the effect of rainfall changes to predict population dynamics of the Asian tiger mosquito Aedes albopictus under future climate conditions
Source: PLoS One. 2022 May 25;17(5):e0268211. doi: 10.1371/journal.pone.0268211 (PMC9132271; doi:10.1371/journal.pone.0268211)
Supplement: Attachment — Submitted filename: Response_to_review_final_20220323.docx [file pone.0268211.s004.docx]

Dear Editor,

Thank you for considering our manuscript entitled “Modeling the effect of rainfall changes to predict population dynamics of the Asian tiger mosquito *Aedes albopictus* under future climate conditions” [Paper # PONE-D-21-35458] for publication in your esteemed journal *PLoS ONE*. We are grateful to the reviewers’ meticulous consideration of our manuscript and their valuable suggestions. We have carefully considered their comments and have implemented all their suggestions through revisions in our manuscript. In addition, we noticed some minor errors in our manuscript and have corrected them (Line 225 Lines 416–417, and caption of Table 3). We apologize for these mistakes. Furthermore, as Fig 4 in the original manuscript had a large file size with an inappropriate resolution (> 10 MB), we divided Fig 4 (a and b) into Fig 4 and Fig 5 and renumbered figures accordingly. Therefore, we have six figures in the revised manuscript.

We hope that our revised manuscript is satisfactory and it is now acceptable for publication.

Sincerely,

Shunji Ohta

**Response to Reviewers’ comment**

**# Reviewer 1**

Thank you for your valuable comments and suggestions. Please note that our replies are included in black font.

1. Only one station data is used to develop the equations. As the insect capture rate is only 1% and depends on habitat meteorological conditions, model developed using one station/location may give a biased output.

**Response 1:**

Thank you for your valuable comment. We agree that your observation that one station can result in a biased output from the PCMP model. However, time-series data on the number of mosquitoes around Tokyo and throughout Japan are not available at this point in time. Therefore, open data published by Tsuda and Hayashi (2014) [39] were used in this study. It is important to validate multilocation results to demonstrate the usefulness of the PCMP model. We have now clarified this aspect by adding content on the relevance of multi-station / location data in the discussion (Lines 384–389). In addition, we propose to consider multi-location data for modeling in the future.

2. The result is, estimated population is over-estimated in autumn and early winter and under-estimated in summer, as the number of observational points is less.

**Response 2:**

Estimation bias is caused by various reasons and one of them is the number of the observation points as suggested by you. Owing to the limitation of accessible data as mentioned above, only one station / location data was used to conduct the PCMP model in this study. Validation using data from multiple stations / locations is an issue that will be addressed in the future. We have added this aspect in Lines 388–389.

3. To predict the population dynamic in future, only one GCM has been used. The projection may be biased for a particular GCM? Why not the authors took multiple GCMs?

**Response 3:**

Thank you for your insightful comment. Comparison of results using multiple GCMs will reveal the general trend for the prediction of mosquito population dynamics. We conducted prediction and projection of mosquito population dynamics using climate data of GFDL and HadGEM based on your advice. The difference between RCP 2.6 and 8.5 on the output of the PCMP model was consistent with that derived using MIROC. It was inferred that the assumption of the responsiveness of the carrying capacity to soil moisture (*v*) will cause a difference in the mosquito population during the active season under future climate conditions. However, the biases in output using inputs from observational meteorology and historical data of GFDL and HadGEM was around 0.3–0.4, which is much smaller than that of MIROC. This implies that the base of the projection is far from the result derived from the observation, leading to unreliable projections in the cases of using climate data of GFDL and HadGEM. Therefore, we retained the results achieved using MIROC shown in the main text and added comments on these results in the discussion (Lines 429–438). We also added the results obtained using GFDL and HadGEM in Supporting Information (S3 Figure, S4 Table, and S4 Text).

4. Isn’t it too early to predict for 2081-2099 as the physiological traits and adapted life cycle of the mosquito will change. What about the near-future/ mid-future projections?

C4:

Based on the inputs provided by your comment, we conducted near-future projections. MIROC climate data during 2031–2049 (both RCP 2.6 and 8.5) were used as inputs for the PCMP model. Differences in the patterns of population dynamics derived from the responsiveness of carrying capacity to soil moisture (*v*) and differences in the output of the PCMP model between the two RCPs were consistent with those obtained in the far-future projection for 2081–2099. However, the projected population sizes at the peak week in the near-future were smaller than those of the far-future because temperature, one of the major driving forces of the PCMP model, in the near-future climate conditions has intermediate values between those under historical and far-future conditions. We added these results in Supporting Information (S4 Figure, S5 Table, and S5 Text) and explained the results in the discussion (Lines 489–495).

**# Reviewer 2**

Thank you for your valuable comment. We present our response below.

A brief description of how the MCMC simulation method was used to estimate PCMP model parameters would clarify calculations.

Thank you for this important suggestion. We have now added a brief description about the MCMC simulation method in Lines 183–187.
