# Supplementary material for: Modeling the effect of rainfall changes to predict population dynamics of the Asian tiger mosquito Aedes albopictus under future climate conditions
Source: PLoS One. 2022 May 25;17(5):e0268211. doi: 10.1371/journal.pone.0268211 (PMC9132271; doi:10.1371/journal.pone.0268211)
Supplement: Peer review history

# Peer review: Decision Letter 0

**DOI:** 10.1371/journal.pone.0268211.r001

**Authors:** Babasaheb B. Fand

7 Feb 2022

PONE-D-21-35458Modeling the effect of rainfall changes to predict population dynamics of the Asian tiger mosquitoAedes albopictus under future climate conditionsPLOS ONE

Dear Dr. OHTA,

Thank you for submitting your manuscript to PLOS ONE. After careful consideration, we feel that it has merit but does not fully meet PLOS ONE’s publication criteria as it currently stands. Therefore, we invite you to submit a revised version of the manuscript that addresses the points raised during the review process.

Please submit your revised manuscript by Mar 24 2022 11:59PM. If you will need more time than this to complete your revisions, please reply to this message or contact the journal office at plosone@plos.org. When you're ready to submit your revision, log on to https://www.editorialmanager.com/pone/ and select the 'Submissions Needing Revision' folder to locate your manuscript file.

Please include the following items when submitting your revised manuscript:

- A rebuttal letter that responds to each point raised by the academic editor and reviewer(s). You should upload this letter as a separate file labeled 'Response to Reviewers'.
- A marked-up copy of your manuscript that highlights changes made to the original version. You should upload this as a separate file labeled 'Revised Manuscript with Track Changes'.
- An unmarked version of your revised paper without tracked changes. You should upload this as a separate file labeled 'Manuscript'.

If you would like to make changes to your financial disclosure, please include your updated statement in your cover letter. Guidelines for resubmitting your figure files are available below the reviewer comments at the end of this letter.

If applicable, we recommend that you deposit your laboratory protocols in protocols.io to enhance the reproducibility of your results. Protocols.io assigns your protocol its own identifier (DOI) so that it can be cited independently in the future. For instructions see: https://journals.plos.org/plosone/s/submission-guidelines#loc-laboratory-protocols. Additionally, PLOS ONE offers an option for publishing peer-reviewed Lab Protocol articles, which describe protocols hosted on protocols.io. Read more information on sharing protocols at https://plos.org/protocols?utm_medium=editorial-email&utm_source=authorletters&utm_campaign=protocols.

We look forward to receiving your revised manuscript.

Kind regards,

Babasaheb B. Fand, PhD

Academic Editor

PLOS ONE

Journal Requirements:

When submitting your revision, we need you to address these additional requirements.

1. Please ensure that your manuscript meets PLOS ONE's style requirements, including those for file naming. The PLOS ONE style templates can be found at

https://journals.plos.org/plosone/s/file?id=wjVg/PLOSOne_formatting_sample_main_body.pdf and

https://journals.plos.org/plosone/s/file?id=ba62/PLOSOne_formatting_sample_title_authors_affiliations.pdf

2. Thank you for stating the following financial disclosure:

“The funders had no role in study design, data collection and analysis, decision to publish, or preparation of the manuscript.”

At this time, please address the following queries:

a)        Please clarify the sources of funding (financial or material support) for your study. List the grants or organizations that supported your study, including funding received from your institution.

b)        State what role the funders took in the study. If the funders had no role in your study, please state: “The funders had no role in study design, data collection and analysis, decision to publish, or preparation of the manuscript.”

c)        If any authors received a salary from any of your funders, please state which authors and which funders.

d)        If you did not receive any funding for this study, please state: “The authors received no specific funding for this work.”

Please include your amended statements within your cover letter; we will change the online submission form on your behalf.

3. In your Data Availability statement, you have not specified where the minimal data set underlying the results described in your manuscript can be found. PLOS defines a study's minimal data set as the underlying data used to reach the conclusions drawn in the manuscript and any additional data required to replicate the reported study findings in their entirety. All PLOS journals require that the minimal data set be made fully available. For more information about our data policy, please see http://journals.plos.org/plosone/s/data-availability.

Upon re-submitting your revised manuscript, please upload your study’s minimal underlying data set as either Supporting Information files or to a stable, public repository and include the relevant URLs, DOIs, or accession numbers within your revised cover letter. For a list of acceptable repositories, please see http://journals.plos.org/plosone/s/data-availability#loc-recommended-repositories. Any potentially identifying patient information must be fully anonymized.

Important: If there are ethical or legal restrictions to sharing your data publicly, please explain these restrictions in detail. Please see our guidelines for more information on what we consider unacceptable restrictions to publicly sharing data: http://journals.plos.org/plosone/s/data-availability#loc-unacceptable-data-access-restrictions. Note that it is not acceptable for the authors to be the sole named individuals responsible for ensuring data access.

We will update your Data Availability statement to reflect the information you provide in your cover letter.

Additional Editor Comments:

Based on the assessment of two anonymous reviewers and my own, I suggest the authors to carry out suggested revisions in their paper

The validity of the model based on single station data needs to be justified.

The details about the modelling procedures and analysis as suggested by reviewer 2 needs to be provided.

The minor language editing to be addressed

[Note: HTML markup is below. Please do not edit.]

Reviewers' comments:

Reviewer's Responses to Questions

**Comments to the Author**

1. Is the manuscript technically sound, and do the data support the conclusions?

The manuscript must describe a technically sound piece of scientific research with data that supports the conclusions. Experiments must have been conducted rigorously, with appropriate controls, replication, and sample sizes. The conclusions must be drawn appropriately based on the data presented. 

Reviewer #1: Partly

Reviewer #2: Yes

**********

2. Has the statistical analysis been performed appropriately and rigorously? 

Reviewer #1: I Don't Know

Reviewer #2: Yes

**********

3. Have the authors made all data underlying the findings in their manuscript fully available?

The PLOS Data policy requires authors to make all data underlying the findings described in their manuscript fully available without restriction, with rare exception (please refer to the Data Availability Statement in the manuscript PDF file). The data should be provided as part of the manuscript or its supporting information, or deposited to a public repository. For example, in addition to summary statistics, the data points behind means, medians and variance measures should be available. If there are restrictions on publicly sharing data—e.g. participant privacy or use of data from a third party—those must be specified.

Reviewer #1: Yes

Reviewer #2: Yes

**********

4. Is the manuscript presented in an intelligible fashion and written in standard English?

PLOS ONE does not copyedit accepted manuscripts, so the language in submitted articles must be clear, correct, and unambiguous. Any typographical or grammatical errors should be corrected at revision, so please note any specific errors here.

Reviewer #1: Yes

Reviewer #2: Yes

**********

5. Review Comments to the Author

Please use the space provided to explain your answers to the questions above. You may also include additional comments for the author, including concerns about dual publication, research ethics, or publication ethics. (Please upload your review as an attachment if it exceeds 20,000 characters)

Reviewer #1: 1. Only one station data is used to develop the equations. As the insect capture rate is only 1% and depends on habitat meteorological conditions, model developed using one station/location may give a biased output.

2. The result is, estimated population is over-estimated in autumn and early winter and under-estimated in summer, as the number of observational points is less.

3. To predict the population dynamic in future, only one GCM has been used. The projection may be biased for a particular GCM? Why not the authors took multiple GCMs?

4. Isn’t it too early to predict for 2081-2099 as the physiological traits and adapted life cycle of the mosquito will change. What about the near-future/ mid-future projections?

Reviewer #2: Title: Modeling the effect of rainfall changes to predict population dynamics of the Asian tiger mosquitoAedes albopictus under future climate conditions

by Shunji OHTA et al.

The authors fitted the physiology-based, climate-driven mosquito population (PCMP) model for temperate mosquitoes to incorporate egg diapauses for hibernating. They also investigated how incorporating the effect of rainfall on larval carrying capacity (into a model) changes the population dynamics of this species under future climate conditions. A comparison was made with rainfall in the model and without rainfall in the model.

This is a well-written paper that addresses an important topic, the population dynamics of mosquitoes in temperate regions. The authors discussed the article in a consistent manner, and it was a pleasure to read. I recommend acceptance of the article for publication in the journal.

Minor revision:

A brief description of how the MCMC simulation method was used to estimate PCMP model parameters would clarify calculations.

**********

6. PLOS authors have the option to publish the peer review history of their article (what does this mean?). If published, this will include your full peer review and any attached files.

If you choose “no”, your identity will remain anonymous but your review may still be made public.

**Do you want your identity to be public for this peer review?** For information about this choice, including consent withdrawal, please see our Privacy Policy.

Reviewer #1: No

Reviewer #2: No

[NOTE: If reviewer comments were submitted as an attachment file, they will be attached to this email and accessible via the submission site. Please log into your account, locate the manuscript record, and check for the action link "View Attachments". If this link does not appear, there are no attachment files.]

While revising your submission, please upload your figure files to the Preflight Analysis and Conversion Engine (PACE) digital diagnostic tool, https://pacev2.apexcovantage.com/. PACE helps ensure that figures meet PLOS requirements. To use PACE, you must first register as a user. Registration is free. Then, login and navigate to the UPLOAD tab, where you will find detailed instructions on how to use the tool. If you encounter any issues or have any questions when using PACE, please email PLOS at figures@plos.org. Please note that Supporting Information files do not need this step.

Attachment Submitted filename: Reviwer Comment PONE-D-21-35458.docx Click here for additional data file.

Attachment Submitted filename: Mosquitoes-Jan 25-2022.docx Click here for additional data file.

# Author comment: Author response to Decision Letter 0

**DOI:** 10.1371/journal.pone.0268211.r002

23 Mar 2022

Response to Reviewers’ comment

# Reviewer 1

Thank you for your valuable comments and suggestions. Please note that our replies are included in black font. 

1. Only one station data is used to develop the equations. As the insect capture rate is only 1% and depends on habitat meteorological conditions, model developed using one station/location may give a biased output. 

Response 1:

Thank you for your valuable comment. We agree that your observation that one station can result in a biased output from the PCMP model. However, time-series data on the number of mosquitoes around Tokyo and throughout Japan are not available at this point in time. Therefore, open data published by Tsuda and Hayashi (2014) [39] were used in this study. It is important to validate multilocation results to demonstrate the usefulness of the PCMP model. We have now clarified this aspect by adding content on the relevance of multi-station / location data in the discussion (Lines 384–389). In addition, we propose to consider multi-location data for modeling in the future. 

2. The result is, estimated population is over-estimated in autumn and early winter and under-estimated in summer, as the number of observational points is less.

Response 2:

Estimation bias is caused by various reasons and one of them is the number of the observation points as suggested by you. Owing to the limitation of accessible data as mentioned above, only one station / location data was used to conduct the PCMP model in this study. Validation using data from multiple stations / locations is an issue that will be addressed in the future. We have added this aspect in Lines 388–389.

3. To predict the population dynamic in future, only one GCM has been used. The projection may be biased for a particular GCM? Why not the authors took multiple GCMs?

Response 3:

Thank you for your insightful comment. Comparison of results using multiple GCMs will reveal the general trend for the prediction of mosquito population dynamics. We conducted prediction and projection of mosquito population dynamics using climate data of GFDL and HadGEM based on your advice. The difference between RCP 2.6 and 8.5 on the output of the PCMP model was consistent with that derived using MIROC. It was inferred that the assumption of the responsiveness of the carrying capacity to soil moisture (v) will cause a difference in the mosquito population during the active season under future climate conditions. However, the biases in output using inputs from observational meteorology and historical data of GFDL and HadGEM was around 0.3–0.4, which is much smaller than that of MIROC. This implies that the base of the projection is far from the result derived from the observation, leading to unreliable projections in the cases of using climate data of GFDL and HadGEM. Therefore, we retained the results achieved using MIROC shown in the main text and added comments on these results in the discussion (Lines 429–438). We also added the results obtained using GFDL and HadGEM in Supporting Information (S3 Figure, S4 Table, and S4 Text).

4. Isn’t it too early to predict for 2081-2099 as the physiological traits and adapted life cycle of the mosquito will change. What about the near-future/ mid-future projections?

C4:

Based on the inputs provided by your comment, we conducted near-future projections. MIROC climate data during 2031–2049 (both RCP 2.6 and 8.5) were used as inputs for the PCMP model. Differences in the patterns of population dynamics derived from the responsiveness of carrying capacity to soil moisture (v) and differences in the output of the PCMP model between the two RCPs were consistent with those obtained in the far-future projection for 2081–2099. However, the projected population sizes at the peak week in the near-future were smaller than those of the far-future because temperature, one of the major driving forces of the PCMP model, in the near-future climate conditions has intermediate values between those under historical and far-future conditions. We added these results in Supporting Information (S4 Figure, S5 Table, and S5 Text) and explained the results in the discussion (Lines 489–495).

# Reviewer 2

Thank you for your valuable comment. We present our response below. 

A brief description of how the MCMC simulation method was used to estimate PCMP model parameters would clarify calculations.

Thank you for this important suggestion. We have now added a brief description about the MCMC simulation method in Lines 183–187.

Attachment Submitted filename: Response_to_review_final_20220323.docx Click here for additional data file.

# Peer review: Decision Letter 1

**DOI:** 10.1371/journal.pone.0268211.r003

**Authors:** Babasaheb B. Fand

26 Apr 2022

Modeling the effect of rainfall changes to predict population dynamics of the Asian tiger mosquito Aedes albopictus under future climate conditions

PONE-D-21-35458R1

Dear Dr. OHTA,

We’re pleased to inform you that your manuscript has been judged scientifically suitable for publication and will be formally accepted for publication once it meets all outstanding technical requirements.

Within one week, you’ll receive an e-mail detailing the required amendments. When these have been addressed, you’ll receive a formal acceptance letter and your manuscript will be scheduled for publication.

An invoice for payment will follow shortly after the formal acceptance. To ensure an efficient process, please log into Editorial Manager at http://www.editorialmanager.com/pone/, click the 'Update My Information' link at the top of the page, and double check that your user information is up-to-date. If you have any billing related questions, please contact our Author Billing department directly at authorbilling@plos.org.

If your institution or institutions have a press office, please notify them about your upcoming paper to help maximize its impact. If they’ll be preparing press materials, please inform our press team as soon as possible -- no later than 48 hours after receiving the formal acceptance. Your manuscript will remain under strict press embargo until 2 pm Eastern Time on the date of publication. For more information, please contact onepress@plos.org.

Kind regards,

Babasaheb B. Fand, PhD

Academic Editor

PLOS ONE

Additional Editor Comments (optional):

Both the reviewers have now recommended to accept the paper based on the author's incorporation of amendments suggested by the reviewers.

I too feel that the paper is now upto the mark to be considered for publication subject to some minor copy editing and grammar/spell check corrections by the authors.

Authors may provide some strong explanations/justification for use of single station data for modelling and simulation. Further to state regarding future line of work on use of multi station data for strong validation of model results

I recommend paper to be accepted after minor connections in the manuscript

Reviewers' comments:

Reviewer's Responses to Questions

**Comments to the Author**

1. If the authors have adequately addressed your comments raised in a previous round of review and you feel that this manuscript is now acceptable for publication, you may indicate that here to bypass the “Comments to the Author” section, enter your conflict of interest statement in the “Confidential to Editor” section, and submit your "Accept" recommendation.

Reviewer #1: All comments have been addressed

Reviewer #2: All comments have been addressed

**********

2. Is the manuscript technically sound, and do the data support the conclusions?

The manuscript must describe a technically sound piece of scientific research with data that supports the conclusions. Experiments must have been conducted rigorously, with appropriate controls, replication, and sample sizes. The conclusions must be drawn appropriately based on the data presented. 

Reviewer #1: Yes

Reviewer #2: Yes

**********

3. Has the statistical analysis been performed appropriately and rigorously? 

Reviewer #1: Yes

Reviewer #2: Yes

**********

4. Have the authors made all data underlying the findings in their manuscript fully available?

The PLOS Data policy requires authors to make all data underlying the findings described in their manuscript fully available without restriction, with rare exception (please refer to the Data Availability Statement in the manuscript PDF file). The data should be provided as part of the manuscript or its supporting information, or deposited to a public repository. For example, in addition to summary statistics, the data points behind means, medians and variance measures should be available. If there are restrictions on publicly sharing data—e.g. participant privacy or use of data from a third party—those must be specified.

Reviewer #1: Yes

Reviewer #2: Yes

**********

5. Is the manuscript presented in an intelligible fashion and written in standard English?

PLOS ONE does not copyedit accepted manuscripts, so the language in submitted articles must be clear, correct, and unambiguous. Any typographical or grammatical errors should be corrected at revision, so please note any specific errors here.

Reviewer #1: Yes

Reviewer #2: Yes

**********

6. Review Comments to the Author

Please use the space provided to explain your answers to the questions above. You may also include additional comments for the author, including concerns about dual publication, research ethics, or publication ethics. (Please upload your review as an attachment if it exceeds 20,000 characters)

Reviewer #1: The review comments have been addressed. In your future work, more number of station data must be used to reduce the bias in results.

Reviewer #2: The authors have addressed all the comments made by referees. Now the paper can be accepted for publication.

**********

7. PLOS authors have the option to publish the peer review history of their article (what does this mean?). If published, this will include your full peer review and any attached files.

If you choose “no”, your identity will remain anonymous but your review may still be made public.

**Do you want your identity to be public for this peer review?** For information about this choice, including consent withdrawal, please see our Privacy Policy.

Reviewer #1: No

Reviewer #2: No

# Editor report: Acceptance letter

**DOI:** 10.1371/journal.pone.0268211.r004

**Authors:** Babasaheb B. Fand

29 Apr 2022

PONE-D-21-35458R1 

Modeling the effect of rainfall changes to predict population dynamics of the Asian tiger mosquito *Aedes albopictus* under future climate conditions 

Dear Dr. OHTA:

I'm pleased to inform you that your manuscript has been deemed suitable for publication in PLOS ONE. Congratulations! Your manuscript is now with our production department. 

If your institution or institutions have a press office, please let them know about your upcoming paper now to help maximize its impact. If they'll be preparing press materials, please inform our press team within the next 48 hours. Your manuscript will remain under strict press embargo until 2 pm Eastern Time on the date of publication. For more information please contact onepress@plos.org.

If we can help with anything else, please email us at plosone@plos.org. 

Thank you for submitting your work to PLOS ONE and supporting open access. 

Kind regards, 

PLOS ONE Editorial Office Staff

on behalf of

Dr. Babasaheb B. Fand 

Academic Editor

PLOS ONE
